# Outcomes of metastatic neuroendocrine carcinoma of the gallbladder

**DOI:** 10.3332/ecancer.2021.1174

**Published:** 2021-01-14

**Authors:** Joydeep Ghosh, Meheli Chatterjee, Sandip Ganguly, Deepak Dabkara, Bivas Biswas

**Affiliations:** Department of Medical Oncology, Tata Medical Center, Kolkata, India

**Keywords:** neuroendocrine, gallbladder, outcomes

## Abstract

**Background:**

Neuroendocrine carcinoma of the gallbladder (NECGB) is a rare pathological entity. They are found to be aggressive cancers. Treatment strategies are based largely on extrapolation from other small cell cancers. Survival is poor compared to adenocarcinoma. Data from low- and middle-income countries are sparse.

**Methods:**

All patients with metastatic NECGB treated in our centre were identified. Their treatment details were captured from electronic medical records. Baseline characteristics were noted and survival was estimated using Kaplan–Meir method.

**Results:**

A total of 15 patients were included. The median age was 55 years. Large cell comprises 2/15 and small cell was found in 13/15 patients. Chemotherapy was platinum-based in 12 patients. The response to first-line chemotherapy was partial in 3 (20%), stable disease in 2 (13.3%) and progressive disease in 10 (66.6%). After a median duration of follow-up of 12 months, the median progression free survival was 3 months and the median overall survival was 5 months.

**Conclusion:**

The outcomes of small cell gallbladder cancer are dismal, despite good response rate. More prospective data are required.

## Background

Neuroendocrine carcinoma of the gallbladder (NECGB) is a rare disease, comprising 0.5% of all gallbladder cancers. They are usually aggressive tumours with poorer outcomes compared to adenocarcinoma. They are mostly found in elderly female patients with underlying cholelithiasis [1]. In a review of 72 cases, the median overall survival (OS) was 13 months. Metastatic disease was present in 72% of the cases [2]. The primary treatment for localised non-metastatic disease is surgery. The role of adjuvant chemotherapy is debated [2, 3]. Most of the advanced cancers are treated with platinum-based doublet regimen, in lines with small cell carcinoma at other sites [4]. Treatment of metastatic disease has led to an improvement in OS [2].

Due to the rarity of the disease, most of the reports have been case reports in the peer-reviewed medical literature. The majority of studies have looked at both localised and metastatic disease together. Here, we report survival outcomes in one of the largest series of patients with metastatic NECGB from a tertiary cancer centre.

## Methods

All patients with a diagnosis of NECGB (grade 3) were identified from electronic medical records in our centre between June 2011 and June 2019 and were included in the analysis. All patients had pathological confirmation of the diagnosis by tissue biopsy. Imaging studies were used to select only those patients who had primary lesion in the gallbladder with/without smaller metastatic lesions in other organs. Patients who received at least one cycle of systemic chemotherapy were included for survival analysis. We studied the clinicopathologic characteristics, treatment received and the outcome. The mitotic index of the tumours was also retrieved from histopathology reports.

All patients had a baseline staging workup with either a contrast-enhanced computerised tomographic scan of the thorax and whole abdomen, or positron emission tomography CT scans or whole body magnetic resonance imaging scan. Baseline performance status and organ function evaluation were evaluated and patients were treated with doublet platinum-based chemotherapy, most commonly etoposide and cisplatin or carboplatin. In those patients with poor performance status, single-agent carboplatin was used. Response evaluation was done at 8–12 week intervals and the response was evaluated as per RECIST criteria.

Survival analysis was done using Kaplan–Meir analysis, using STATA version 14 (StataCorp LLC 4905 Lakeway Drive College Station, TX, USA) [6]. Univariate or multivariate analysis was not done due to very small numbers. OS was calculated from the date of the start of treatment to date of death due to any cause. Progression-free survival (PFS) was calculated from the date of diagnosis to the date of progression/death due to any cause. The data were censored on 1 July 2020.

## Results

A total of 15 patients were identified with a median age of 55 years (range: 40–73) and a male:female ratio of 1:4. Amongst subtypes, large cell NEC subtype was seen in 2 patients and small cell in 13. The mean mitotic index was 70% (range 40%–90%). The most common sites of metastasis were liver in 8 (53.3%), followed by abdominal lymph nodes in 4 patients, and lung, bone and omentum were the sites of metastasis in 1 patient each. Three patients had a past history of open cholecystectomy before they came to the hospital. The post-operative specimens of these three patients were as follows: pT2: two patients, pT3: one patient, pN1: all three patients. All three patients had metastatic disease. Chemotherapy was platinum-based in 12 patients (single-agent carboplatin in three, etoposide-carboplatin in nine) and non-platinum in 3 patients (capecitabine for two patients and somatostatin long acting for one patient). The response to first-line chemotherapy was partial in 3 (20%), stable disease in 2 (13.3%) and progressive disease in 10 (66.6%). The clinicopathological characteristics are depicted in Table 1.

**Figure 1.**
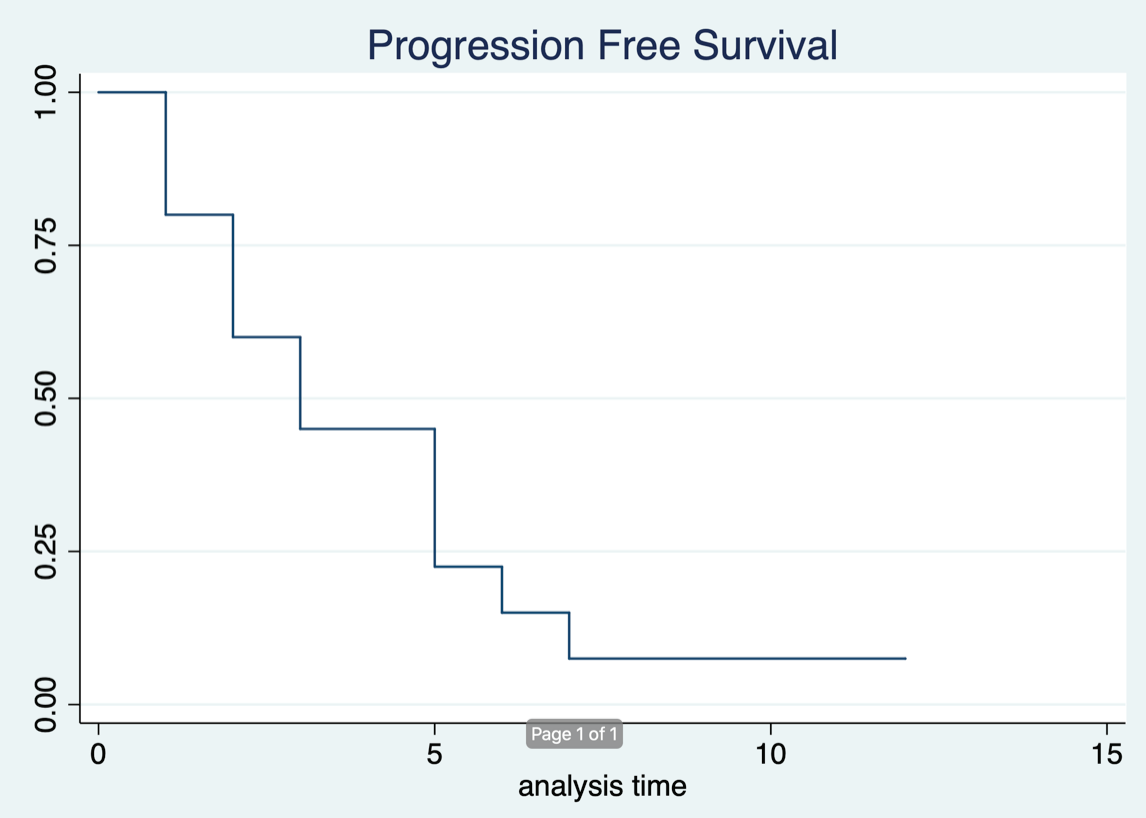
Progression free survival.

**Figure 2.**
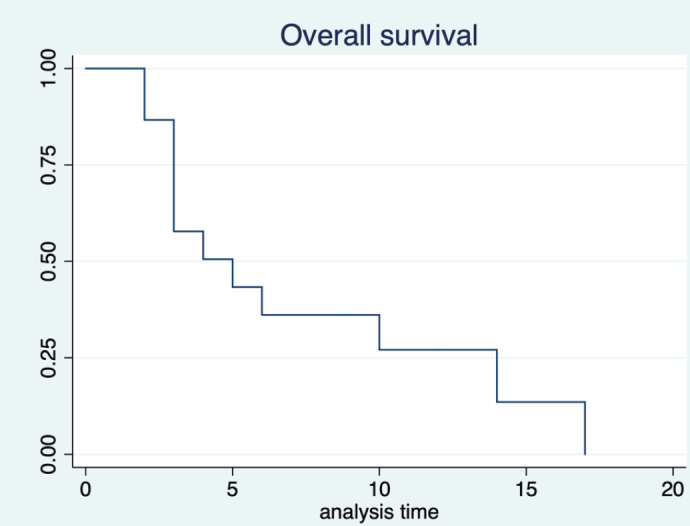
Overall survival.

The median duration of follow-up was 12 months (95% CI: 9months–not reached). Out of 15, 13 patients have had progression. The median PFS was 3 months (95% CI: 1–5 months). The 6 month PFS was 14.7% (95% CI: 2.4%–37.4%) (Figure 1). Twelve out of 15 patients died, all the deaths were due to disease progression. The median OS was 5 months (95% CI: 3–14 months). The 12 month OS was 26.9% (95% CI: 7.3%–51.6%) (Figure 2).

## Discussion

NECGB is a very rare entity. The reported incidence is 0.02% (21 per 100,000 population) as per the SEER database [7]. A large proportion of these cases (90%) were not staged at diagnosis [7]. Neuroendocrine tumour of gallbladder accounts for less than 0.5% of all malignant tumours of gallbladder [8]. It is usually classified into four major categories based on tumour grade and mitotic index: well-differentiated neuroendocrine tumour (typical carcinoid), well-differentiated NEC (atypical carcinoid), poorly differentiated NEC (small or large cell) types and mixed exocrine–endocrine carcinomas. Sometimes there can be both the components of adenocarcinoma and neuroendocrine carcinoma (MANEC) [9]. Here in this study, we have analysed only the poorly differentiated NEC (small or large cell type).

The median age of presentation is usually in the range of 60–70 years [2, 7, 9]. Female individuals are more commonly affected than males, which is 80% of this population. Patients usually present at an advanced stage with 70% of cases being metastatic [2]. The median age of study population was younger at 55 years. The most common clinical presenting symptoms were abdominal pain, jaundice, abdominal mass, weight loss, poor appetite and weakness [5, 7, 10]. The definite diagnosis is made by histopathological examination and immunohistochemistry staining. The tumours are usually positive for chromogranin, synaptophysin and CD 56A [11, 12]. They can be classified into subtypes based on the mitotic index. A mitotic index of more than 20% is usually suggestive of high-grade histology that differentiates it from low-grade NETs [13]. Since we have analysed only high-grade NEC, all our patients had a mitotic index of more than 20%, with a mean of 40%. The liver was the most common site of metastasis for these cancers, followed by abdominal and retroperitoneal lymph nodes [8, 11, 13]. This distribution of metastasis was also seen in our study, with liver as the most common site of metastasis. Lung and bone metastasis is quite rare, and our series had just one each [14, 15].

Treatment of these cancers is usually an extrapolation of treatment strategies of small cell carcinoma of the lung. It involves a platinum-based doublet regimen, most commonly etoposide and cisplatin or carboplatin [16–20]. In our series, the majority of the patients, 12 (80%) received a platinum-based regimen. The median PFS in our study was 3 months. Long-term survival after platinum-based doublet has rarely been reported in the literature. The OS of this population was 5 months. This indicates that after progression, these patients usually do not survive long, as there are no standard second-line options available.

## Conclusion

In conclusion, our study represents one of the largest ones of NECGB. The limitations of the study were the retrospective nature of data capture. Although the sample size is small at 15 patients, we feel our data are important, considering the rarity of the entity. A prospective multi-centre collaborative study in a larger sample size is needed in this rare entity for better insight into the natural history of these diseases and their treatment outcomes.

## Conflicts of interest

None.

## Funding

Nil.

## Authors’ contributions

Concept and design: JG, MC, SG. Data entry, analysis: JG, BB, SG, MC. Manuscript writing, proofreading, final approval: all authors.

## Figures and Tables

**Table 1.** Baseline characteristics.

Age	Median: 55 years	Range 40–73 years
	*N*	**%**
Gender	Male: 3 Female: 12	20 80
Sites of metastasis	Liver alone: 8 Abdominal lymph nodes: 4 Omentum: 1 Lung: 1 Vertebra: 1	53.3 26.6 6 6 6
Microscopic subtype	Large cell neuroendocrine variant: 2 Small cell neuroendocrine variant: 13	13.4 86.6
Mitotic count	Median 70%	Range 40%–90%
Past surgical history	Surgery done: 3 No surgical history: 12	20 80
Chemotherapy regimen	Platinum: 12 Non-platinum: 3	80 20
Chemotherapy cycles	Less than 3: 5 3 or more: 10	33.3 66.7
Overall response:	Partial: 3 Stable: 2 Progressive: 10	20 13.3 66.7
